# High pathogenicity avian influenza virus emergence: Blame it on chickens or on humans raising chickens?

**DOI:** 10.1371/journal.ppat.1012608

**Published:** 2024-10-16

**Authors:** Juliette Gross, Romain Volmer, Pierre Bessière

**Affiliations:** Ecole nationale vétérinaire de Toulouse, Université de Toulouse, ENVT, INRAE, IHAP, UMR 1225, Toulouse, France; Mount Sinai School of Medicine, UNITED STATES OF AMERICA

## Introduction

Low pathogenicity avian influenza viruses (LPAIV) of H5 and H7 subtypes can naturally evolve to replicate systemically in birds, acquiring a high pathogenicity avian influenza virus (HPAIV) phenotype [1]. As well as having a considerable impact in veterinary medicine, HPAIVs jeopardize food safety and public health because of their zoonotic potential. A number of genetic mechanisms have been identified as being responsible for the evolution from LPAIV to HPAIV. The determinant change is the acquisition of a furin-like protease-dependent hemagglutinin multibasic cleavage site (MBCS), via non-synonymous nucleotide substitutions, nucleotide insertions, or recombination with cellular or viral RNAs caused by viral polymerase template switching. In addition, other mutations in the hemagglutinin and other viral gene segments are sometimes required for the acquisition of a high pathogenicity phenotype [2]. The molecular mechanisms underlying genetic changes leading to HPAIV emergence, including its restriction to H5 and H7 subtypes, will not be discussed further, as the aim of this article is to provide a multi-faceted analysis of the role of the host in the emergence of HPAIV. It is commonly accepted that conversion from LPAIV to HPAIV occurs in terrestrial poultry belonging to the Galliformes order, particularly in chickens and turkeys. In the following sections of this article, we will describe supporting evidence for a decisive role of Galliformes in HPAIV emergence, and also delineate the factors that could promote HPAIV emergence in Galliformes compared to other bird species.

## HPAIV emergence versus first detection

A total of 47 LPAIV to HPAIV conversion events were recorded between 1959 and 2024 worldwide [3,4]. On 43 occasions, the newly emerged HPAIV strain was first detected in terrestrial birds, and 41 HPAIV were first detected in Galliformes, more specifically 33 in chickens and 8 in turkeys. The remaining 2 HPAIV first detected in terrestrial birds originated from ostriches. On 4 occasions, HPAIV were first isolated from water birds: in 1961 in wild terns belonging to the Charadriiformes order [5], in 2015 from fattening duck farms [6], and in 2021 [4] and 1996 [7], respectively, in swans and geese belonging to the Anseriformes order. The latter corresponds to the first detection of the goose/Guangdong-lineage of H5 HPAIV lineage that subsequently spread to countless poultry farms and wild birds around the world [8]. The proportion of HPAIV first detected in chickens is therefore 70% compared with 17% in turkeys and 4% in ostriches, making chickens the species in which the majority of HPAIV isolates were first found.

Water birds of the Charadriiformes and Anseriformes order are the main reservoirs of LPAIV [1], allowing LPAIV to perform more infections and altogether more replication cycles in water birds than in terrestrial birds: this should, theoretically, give LPAIV more opportunities to evolve in water birds. A blunt interpretation of the above reported figures would thus be that HPAIV are more likely to emerge in terrestrial birds than in water birds. In addition, evidence suggests that the mutation rate is higher in avian influenza A viruses infecting chickens than in those infecting ducks, providing further support to this assertion [9]. However, it is important to distinguish LPAIV to HPAIV conversion events and the first detections of HPAIV, which provide the name to the strain and consequently become eventually the only traceable element. Detection of newly emerged HPAIV by passive surveillance may be more likely in Galliformes because these birds typically have a more severe clinical presentation than Anseriformes [1], which may spread HPAIV undetected. Conversely, although the goose/Guangdong-lineage of H5 HPAIV was first detected in geese, this does not necessarily mean that the low to highly pathogenic conversion took place in geese. Consequently, the fact that the vast majority of first HPAIV detections have occurred in chickens does not necessarily mean that LPAIV to HPAIV conversion occurred in this species.

## Epidemiological evidence for HPAIV emergence in terrestrial birds

Taking into account these limitations, only few studies provide strong epidemiological evidence regarding the role of a specific bird species in HPAIV emergence, because the LPAIV progenitor was detected in the same or neighboring farm prior to HPAIV detection (**Table 1**) [10]. The 15 LPAIV to HPAIV conversion events meeting the above-described epidemiological criteria all occurred in terrestrial birds (chickens, turkeys, or ostriches), supporting the notion that HPAIV may be more prone to emerge in terrestrial birds than in aquatic birds.

**Table 1:** High pathogenicity avian influenza viruses for which the low pathogenicity progenitors were detected in the same or neighboring farms prior to HPAIV detection.

Year	Country	Subtype	HA accession numbers	Progenitor HA accession number	Species (first detection)
1966	Canada	H5N9	CY107859	CY087808	Turkeys
1976	Australia	H7N7	CY024786	CY061602	Chickens
1983–1984	USA	H5N2	GU052771	J04325	Chickens
1994–1995	Mexico	H5N2	AB558473 U85390	GU186573	Chickens
1999–2000	Italy	H7N1	CY021405	GU052999	Turkeys
2002	Chile	H7N3	AY303631 AY303632	AY303630	Chickens
2003	Netherland	H7N3	EPI1595425	EPI1595417	Turkeys
2004	Canada	H7N3	AY648287	AY650270	Chickens
2006	South Africa	H5N2	EF591749	EF591757	Ostriches
2008	United-Kingdom	H7N7	FJ476173	Partial sequence in [19]	Chickens
2015	United-Kingdom	H7N7	EPI623939	Partial sequence in [20]	Chickens
2015	Germany	H7N7	EPI634885	EPI624526	Chickens
2016	USA	H7N8	KU558906	EPI709576	Turkeys
2017	USA	H7N9	MF357740	MF357732	Chickens
2020	USA	H7N3	EPI1775733	MT444363	Turkeys

## Weak molecular evidence for emergence in chickens

Acquisition of an MBCS via recombination/template switching with host genes has been described on multiple occasions after an infection with a H7 virus [11]. In 4 natural HPAIV emergence events, the identity of the host gene was identified as ribosomal RNA (rRNA) of chicken origin [12]. Yet, this molecular signature does not unambiguously mean that the LPAIV to HPAIV conversion occurred in chickens, as rRNA sequences are highly conserved [13]. Indeed, in all 4 cases, it is easy to find a perfect match of the inserted sequence not only with chicken rRNA, but also with rRNA of birds belonging to various orders, including Anseriformes (**Table 2**). Knowing this, the search for the origin of the inserted rRNA sequences needs to be broadened to other species and complemented with epidemiological investigations.

**Table 2:** Recombination between HA and 28S ribosomal RNA could have occurred in bird species other than chickens. By blasting the sequence corresponding to the original ribosomal insert, the alignment indicates a perfect match not only with 28S rRNA sequences from chickens, but also a perfect match with 28S rRNA from other birds.

Strain	HA cleavage site sequence with 28S insert sequence underlined (*amino acid sequence*)	Perfect match with 28S rRNA from a selection of bird species (NCBI accession number provided)
A/chicken/SK/HR-00011/2007(H7N3)	CCCAGAGAACCCCAAGACCACGAAACCCCGACCCAGAAGAGGCCTTTTT (*PELPKGTKPRPRRGLF*)	**Chicken** (XR_006936395), **Common eider** (OX598325), **Ruff** (XM_014959211), **White-tailed eagle (OX381649), Eurasian curlew** (OZ067259), **Common goldeneye** (OZ060841), etc.
A/chicken/Spain/6279–2/2009(H7N7)	CCCGAACTCCCAAAGGGAACGAAACCCCGACCCAGAAGAGGCCTATTT (*PELPKGTKPRPRRGLF*)	**Chicken** (XR_006936395), **Tufted duck** (XR_004254457), **Common goldeneye** (OZ060841), **Red-crested pochard** (OZ037822), **Long-tailed duck** (OZ022461), **Northern pintail** (OZ009993), etc.
A/chicken/Jalisco/CPA1/ 2012(H7N3)	CCAGAGAACCCCAAGGATAGGAAGAGCCGACATCGAAGGACCAGAGGCCTTTTT (*PENPKDRKSRHRRTRGLF*)	**Chicken** (XR_006936395), **Red-crested pochard** (OZ037822), **Pink-footed goose** (OZ035909), **Emu** (XR_010387072), **Great cormorant** (XR_010376445), **Long-tailed duck** (OZ022461), etc.
A/chicken/Tennessee/17–007147-2/2017(H7N9)	CCAGAGAACCCCAAGACCGATAGGAAGAGCCGACATCGAAGGATCAGAGGTCTTTTT (*PENPKTDRKSRHRRIRGLF*)	**Chicken** (XR_006936395), **Common goldeneye** (OZ060867), **Rock dove** (XR_010468096), **Red-crested pochard** (OZ037822), **Pink-footed goose** (OZ035909), **Great cormorant** (XR_010376445), etc.

## Experimental evidence supporting a higher propensity of HPAIV emergence in chickens compared to ducks

Acquisition of an HA MBCS is due to viral polymerase induced mutations or template-switching occurring during the replication of an LPAIV progenitor. Consequently, HPAIV appear in a host already infected with an LPAIV. The direct implication thereof is that the LPAIV precursor and its HPAIV descendant coexist in the index host where the HPAIV has evolved [14]. The chances of successful HPAIV emergence, defined here by the ability to be transmitted to new individuals, are therefore likely influenced by the nature of the interaction between the minor HPAIV variant and its LPAIV precursor. In an LPAIV HPAIV coinfection model in chickens, the HPAIV to LPAIV ratio in the inoculum was shown to determine the probability of the HPAIV to be selected over the LPAIV [15]. Subsequently, LPAIV HPAIV coinfections were carried out in chickens and ducks to determine if the host species modulated the within-host interaction between the HPAIV and LPAIV. In an H5 LPAIV HPAIV coinfection model, HPAIV selection was significantly more likely in chickens than in ducks [16]. These results were later confirmed with a pair of H7 LPAIV and HPAIV [17]. While the reasons for the species-specific differences in the interaction between the LPAIV and HPAIV remain to be determined, altogether these two studies were the first to provide experimental evidence that HPAIV may be more prone to emerge in chickens than in ducks.

## Taking a bird’s eye view on HPAIV emergence: The impact of farming

The fact that the number of chickens by far exceeds that of other bird species also likely contributes to the overrepresentation of chickens in HPAIV emergence events. Chicken populations have grown steadily: By 2020, there were more than 33 billion of them on Earth, making them the world’s most farmed animals, compared to approximately 1 billion farmed ducks [18]. As farms can contain hundreds of thousands, if not millions of chickens, they are ideal infrastructures for viral evolution. When an LPAIV precursor is introduced into a farm, it can successively infect numerous individuals, encouraging the emergence of more transmissible variants, hence giving HPAIV an even stronger selective advantage in densely populated farms than in the wild. Thus, HPAIV could emerge preferentially in chickens, not solely for reasons intrinsic to this species, but also because of farming conditions.

## Conclusion

HPAIV emergence is a question of a combination of probabilities. The first is the probability of an LPAIV to acquire an MBCS in the HA, which we hypothesize to be mainly dependent on virological features not discussed here, as the observation that viruses may have a higher mutation rate in chickens than in ducks remains to be experimentally proven [9]. The second is the probability of the newly formed HPAIV to be selected over the parental LPAIV within the index host to be transmitted to a naïve individual. The third one is the probability to sustain interindividual transmission to initiate and maintain an epizootic. The last 2 probabilities are likely dependent on a combination of host-species intrinsic properties, such as their innate immune response, or the expression of specific receptors and their tissue distribution, and man-made ecological modifications, such as the organization of farming or global landscape and climate changes. Ultimately, chickens appear to be the prominent species responsible for HPAIV emergence, but may be retrograded to second place behind turkeys if we normalize the number of emergence events attributed to a specific species by the number of birds of each species. Similarly, an apparent lower probability of HPAIV emergence in Anseriformes does not mean that emergence in Anseriformes is impossible. Further work aiming at understanding the factors that modulate HPAIV emergence is required to help prevent future devastating epizootics at its root, before these get out of control.
